# Cell surfaces.

**DOI:** 10.1038/bjc.1966.38

**Published:** 1966-06

**Authors:** M. R. Anderson


### CELL SURFACES

MARY R. ANDERSON

*From the Department of Experimental Pathology and Cancer Research, School of Medicine, Leeds 2*

Received for publication March 11, 1966

LITTLE is known about the cell surface. Until recently it was thought to be only the coating of the cell, just as the skin was the envelope of the body, and was believed to be only of structural significance. Recently the skin has gained status as an organ, and the cell surface is now under detailed investigation.

Robertson (1960) investigated the structure of the cell membrane and the endoplasm, which he found to be a continuous reticulum of the cell. From his electron microscope studies he has suggested a compound membrane of four layers, which may consist of a protein monolayer on one side of a lipid core, and perhaps carbohydrate on the other side, in some cases ; Dourmashkin, Dougherty and Harris (1962) have demonstrated electron microscope studies of red blood cells which show plaques 40 Å by 250 diameter, composed of lecithin and cholesterol with protein fibres 20 by 100–200 Å long lying beneath the plaques. There is also found to be a surface area of the cell which consists of a thin layer of cytoplasm which is a poor conductor of electricity (Jacobs, 1962), while the interior of the cell is a good conductor. Also substances which are unable to diffuse across the cell membranes, diffuse readily if they are injected into the cell (Chambers, 1922).

The surface membranes are not homogeneous, but have specialised areas with regulators of the cell uptake lying in, or on, the cell surface (Edelberg, 1952). There appears to be a surface preference for lipid-soluble substances, (Robertson, 1960) ; Van der Waals forces are thought to hold lipid molecules together to protein side chains.

Pulvertaft (1946) has shown rhythmic movement or flicker on red cells, and Weiss (1965) has suggested recently that the *in vivo* cell undergoes continuous modification by sublethal autolysis, and that this autolysis is under endocrine control. In support of this he has shown that rat fibroblasts treated with antiserum lose their acid phosphatase staining and become detached from glass surfaces, and that both the loss of staining and tendency to detach are retarded by hydrocortisone.

Rosenberg (1964) concurs with Weiss, and says the cell in equilibrium with the environment modifies itself according to changes in the environment. He grew human conjunctival cells at the interface between two liquids, one hydrophobic and one hydrophilic, and found the cells grew in layers. However, when he added lecithin, the cells grew in clumps.

Similarly, Sawant, Desai and Tappel (1964) have demonstrated binding of enzymes to different sites on lysosomal membranes, and Bullough (1966) has described feedback systems controlling the synthesis and activation of enzymes by repressor molecules binding metabolites which, he believes, activate or inactivate the repressor.

Desai and Glover (1963) believe there is an association between the lipid components of cell membranes and the permeability of the membranes, the phospholipid content of which is genetically regulated and varies with the species. The surface substance saponin or lysolecithin, which is produced by intracellular phospholipase from lecithin, causes minute perforations in lipid membranes. Bimolecular membranes may be damaged also by an accumulation of surface-active anions, especially fatty acids which cause emulsification of the membranes. It is thought the membranes may be reconstituted by the incorporation of surface-active cations ; Bangham, Rees and Shortlander (1962) have prevented liver necrosis in rats subjected to carbon tetrachloride by the i.p. injection of cetyltrimethylammonium bromide, which supports this hypothesis.

Lipids, too, play a complicated role in the functional activity of cell membranes. On the one hand, they permit the passage of lipid-soluble drugs, while on the other hand, they assist in maintaining the potassium balance of the cell (Coburn, 1963). Shaprir and Kerpel (1964) have shown that triglycerides are in a state of balanced synthesis which is under hormonal control, and Hotchkiss (1944) showed that lipids protect the cell against metabolic effects of noxious agents which blocked the potassium uptake in bacteria, and demonstrated that this is prevented by cephalin; hence Gram-negative bacteria which contain cephalin are protected from the effects of gramicidin. Similarly streptolysin inhibitors are associated with A and B lipoproteins.

Disulphide bonds are also thought to play a part in the structure of gamma globulin by linking the A and B chains (Cohen and Porter, 1964), and are believed to be involved in the biological activity of proteins and peptides. Indeed the activity of the hormone vasopressin in the toad has been shown by Schwarz (1959) to depend on the presence of SS bonds, as the influence of the hormone in increasing the permeability of the toad bladder is abolished if the SS bonds are opened by SH reagents. Insulin is also believed to be similarly dependent on the SS bond according to Rasmussin, Schwartz and Schoessler (1962) : indeed it is thought that all disulphide containing hormones have similar properties, and other workers have isolated protein-steroid complexes and explored their antigenic properties, and it is believed that the protein steroid complexes may act as haptens, possibly with steroid specificity.

Kidson and Kirby (1964) believe that synthesis of RNA is under hormone control, and that induction of enzyme synthesis by cortisone is regulated at the level of DNA transcription to messenger RNA. They found they could get altered (and reversible) patterns of messenger RNA by either starvation or by the use of hormones ; most of the hormones they tested altered messenger RNA selectively, increasing some fractions and diminishing others. They think that the hormones act on genes by direct effect on the DNA-protein complex, possibly interacting with repressor proteins, and that rapidly labelled RNA may act as repressor at either the DNA or the ribosomal level. Sekeris and Karlson (1964) also think that hormones activate the genes which produce messenger RNA, which in turn leads to the synthesis of specific proteins. They found that the hypothesis, that inhibitors of nucleic acid and protein biosynthesis also inhibit the activity of hormones, is true for the larvae of *Calliphora.* Liao and Williams-Ashman (1962) showed that the first effect of hormone is the production of messenger RNA, as the action of the hormone is abolished by inhibitors of protein synthesis.

It is however unwise to over particularise regarding the action of hormones, in the light of the findings of Michael (1964) who investigated the distribution of oestrogen after s.c. injection in the cat, and found uptake not only by the genital tract but also by the pituitary, from where it localised in the septum and hypothalamus. Nelson *et al.* (1964) have studies the hormonal steroids, and find that a number of investigators (Taliaferro, Cobey and Leone, 1956 ; Wallace, Silverberg and Carter, 1957 ; Slaunwhite and Sandberg 1959) have demonstrated the effect of corticosteroid-binding globulin on the metabolism of the corticosteroids in pregnancy—or patients receiving oestrogen, who have an increase in the binding proteins in the circulation with a resultant doubling or tripling of basal levels of measurable corticosteroids. Despite the measurable levels of corticosteroid, however, the material is thought to be biologically inactive in a bound state. Nelson *et al.* (1964) conclude that oestrogens in human blood are in several forms, mainly oestrogen sulphate, but there are also glucosiduronate conjugates, and sulphoglucosidurate double conjugates, as well as free oestrogens. They found that conjugation can occur in various tissues, which included foetal tissue as well as the liver and intestinal mucosa.

On the other hand, Cope (1965) who has worked on adrenal steroids, has found that they have a variable effect related to the concentration in the blood, the metabolic state, and whether the steroids are in a protein-bound or free state. He has recently suggested there may be separate binding sites for each steroid, and that only the free steroid is normally active, and he believes that binding delays steroid diffusion across membranes. Other workers (Erlanger *et al.*, 1957 ; Beiser *et al.*, 1959) believe that protein-steroid complexes can act as haptens. In this connection the work of Movat and Fernando (1963) is relevant. They found complexes formed without cell-binding, which can cause cellular injury and induce an inflammatory response. It is possible therefore that some ranges of hormonal imbalance may act as cellular irritants.

Riggs (1964) considers that hormones may enter cells by crossing the membranes in a carrier state, and may be released again within the cell. He also believes hormones can alter membranes to permit nutrients to enter. In support of this, it is known that sodium moves out of a cell in response to insulin.

According to Coutinho (1965) many investigators believe that the mechanism of action of steroid hormones is related to cytoplasmic enzyme-substrate reactions, and steroid hormonal effects have been demonstrated *in vitro* in several enzymatic reactions (Villee, 1959 ; Engles, 1959 ; Talalay and Williams-Ashman, 1960), although no clear connection between these effects and the physiological role of the hormones has been firmly established.

It has been shown by Csapó (1960) that modifications in the ionic environment profoundly alter the properties of the excitable membrane of the uterine cell, and the excitation process, which triggers the myoplasmic machinery, can be reversibly suppressed. It was also shown that the ovarian steroids alter the ionic balance of the myometrial cell, and that such alterations modify its excitability and pharmacological reactivity. Thus, it was proposed that the primary target of the ovarian steroids is the myometrial cell membrane, and that the action of these regulating agents is mediated by ions (Csapó, 1956).

This relationship between steroid hormones, ions and membrane function operates not only in the regulation of myometrial function, for there is accumulating evidence (Tepperman and Tepperman, 1960) that other steroid hormones also profoundly modify the ionic balance in mammals.

Ions activate enzyme systems, and it is known that both calcium and magnesium play a prominent role in carbohydrate, protein, and lipid synthesis. Indeed the balance between these two ions may regulate the reaction rates and, as Dixon and Webb (1958) have suggested, ovarian hormones may act by changing the ionic state within the cell. They found that one of the results of oestrogen treatment was an increase in divalent cations, especially calcium, in the cell which, according to them, explains both the anabolic and the stimulating effect of the hormone. They also found that progesterone prevented calcium entering the cell, and magnesium dominated both the functioning of the cell membrane and the metabolism of the cell. Heilbrunn (1956) showed that calcium alters the viscosity of protoplasm when it enters a cell quickly, and increases the firmness of the cortical area of the cell. Gent, Trounce and Walser (1964) have demonstrated sites on human red cells which bind calcium ions. These sites are believed to be either polyvalent, or a regular array of singly charged groups lying close together. They are thought to be phosphate groups of phospholipids or polyphosphoinositides with sialate groups on the surface of the cell.

According to Jensen (1963) the three primary steroid sex hormones, oestradiol, progesterone and testosterone, are only metabolised by the non-responsive tissues which have a special affinity for them. As steroid oestrogens have a very high affinity for some proteins, it is thought they may bind to $\Delta^5$ steroid isomerase an hydroxysteroid dehydrogenases. Hormones could therefore not only exert a positive or negative action by blocking growth-restraining factors, but they could accelerate enzyme function, or alter nuclear or cell membrane permeability, by causing calcium binding.

The possibility should therefore be considered that hormones may have varying effects according to the number of specificities which they have available for interaction with a cell. Similarly, a cell under antibody attack could presumably respond atypically to a hormone if a number of the reactive cell sites were already blocked by the antibody. Indeed Weir and Pinckard (1965) have already demonstrated complement fixing antibodies against the mitochondria of rat liver which had been previously damaged by carbon tetrachloride. These antibodies were found to be transient, and it is not known whether they contribute to tissue damage or are the result of an immune response to the damage; but whether they are the result or the cause of the tissue damage, they could theoretically greatly modify the response of the cell to hormonal regulators.

Several variations from comparable normal cells have been described in neoplasia. Rapport and Graf (1961) have found that tumour tissue contains phospholipid haptens which are not found to any extent in non-malignant tissues. They also obtained an antitumour gamma globulin which they demonstrated by radio-active iodine, and found that complement and the specific antibody caused a loss of free amino acid ribonucleotides and potassium from the target cell.

Glick and Githens (1964) have shown that tumour cells concentrate potassium ions more efficiently than normal cells, and that removal of the sialic acid from the cell surface inhibits this uptake.

Kalchar (1964) has found 4 types of malignant cells with a diminished capacity to convert glucose to galactose compared to similar normal tissues. He believes that the ability to synthesis galactose compounds may be insufficient to maintain cell surface polysaccharides, and this results in a loss of surface antigens in the neoplastic cells.

Electrophoresis has been used to study the structure of the cell surface. Fuhrmann (1965) has worked with isolated liver parenchymal cells of various inbred rat strains, and found little or no difference in the electrophoretic mobilities. He found the electrophoretic mobility of rat liver proliferating after partial hepatectomy correlated with the increased mitotic index which is found during liver regeneration. However, it did not correlate with the diurnal variation in normal liver mitotic index. He also found that when he treated proliferating liver cells with neuraminidase the electrophoretic mobility was unchanged. When, however, he treated hepatoma cells with neuraminidase there was a 50% reduction in the mobility which, he claimed, is evidence of the structural alteration in the malignant cells. His findings may be summarised in his own words :

1. " Proliferating as well as malignant liver cells of Wistar rats have higher electrophoretic mobilities than the normal liver cells of the same strain.

2. " A definite difference in electrophoretic mobility between proliferating liver cells and malignant liver cells is found after treating both cell types with neuraminidase ; in both cases neuraminic acids are in the supernatant.

3. " After transferring the malignant liver ascites from Wistar to Sprague-Dawley and BAL rats, the malignant cells retain the electrophoretic characteristics of the donor strains.

4. " *In vivo* neuraminidase treatment of rats harbouring a malignant liver ascites and mice harbouring an Ehrlich ascites tumour, as well as *in vitro* treatment of Hela-cell cultures affects neither the *in vivo* or the *in vitro* viability of these tumour cells. "

Fuhrmann (1965) also found a variable between normal tissues after treatment with neuraminidase. Rat kidney cells had a markedly increased electrophoretic mobility, whereas liver cells only showed a slight increase. He considers this is due to secondary changes which may not occur as a result of the enzyme treatment. Working on the same problem, Doljanski and Eisenberg (1965) concluded from their experiments that the increase of mobility observed in both malignant and regenerating cells was not directly associated with cell division.

To clarify this question, the electrophoretic mobility of synchronised cultures of *Escherichia coli B.* was examined in the laboratory of Schulman (1965). It was found that cell samples, representing different stages in the mitotic cycle, all had the same electrophoretic mobility. This observation is in line with findings of Ruhenstroth-Bauer *et al.* (1962), according to which regenerating liver cells exhibit the same increased mobility when measured during the troughs or the peaks of the mitotic cycle. Similarly, measurements of Ehrlich ascites tumour cells, even when effected in various phases of mitosis, showed a very small scatter in mobility values (Cook, Heard and Seaman, 1962). Furthermore, as demonstrated by Forrester, Ambrose and Macpherson (1962), polyoma induced malignant cells manifested an increased charge when compared with normal cells with the same rate of growth.

All these observations lead to the conclusion that the increased mobility observed both in cells undergoing a regenerative growth process, as well as in malignant cells, is not associated with the cell division cycle as such. The possibility must be considered whether this increased mobility could be due to a temporary increase of surface charge in the case of regenerating cells, and a permanent increase in malignant cells. Such an alteration in charge may be associated with an increased work potential, and an alteration in membrane permeability. The alteration in permeability, if it occurs, would favour both regenerative and malignant tissues as compared with normal cells in competition for nutrients.

These studies support the growing evidence of cell membrane involvement in carcinogenesis, and study of the cytoplasm and its membranes in the development and growth of cancer has been given additional impetus by the findings of McKinnell (1962) who has worked on the eggs of the frog *Rana pipiens.* He activated the eggs, and removed the nucleus which he replaced with the nucleus from disassociated renal adenocarcinoma cells from the Lucke tumour of frogs. The experimental eggs developed to abnormal swimming forms, while the control eggs transplanted with normal blastula nuclei obtained complete metamorphosis. The interesting thing in these experiments is that a degree of organised growth was obtained with malignant nuclear transplants. If the fundamental lesion in cancer were nuclear, a mass of disorganised tissue would have been the expected result of these transplant operations.

In tissue culture at any rate, direct contact between cells seems to exert some effect, for Moller (1965) has shown that immune lymphocytes containing histocompatible antigens of the $F_2$ type exert a marked cytotoxic effect on tumour cells incompatible with the $H_2$ antigen. Also when he added either heat inactivated rabbit serum or phytohaemagglutinin to a mixture of tumour cells, and either allogeneic or semiallogeneic lymphocytes of $F_1$ hybrids which caused the lymphocytes to aggregate around the tumour cells, he again obtained death of the tumour cells, hence close contact with histoincompatible lymphocytes results in death of the tumour cells.

Evidence is slowly mounting that antibody attack can so modify the cell that it responds atypically to hormonal regulators. If protein-steroid complexes can also act as cell irritants and induce an inflammatory and antibody response, there is a further possibility of atypical functioning of the cell membrane.

Allison, Smith and Wood (1955) have studied the effect of cortisone on the cellular response to thermal injury, and concluded that cortisone exerts a direct protective action on endothelial cells and leucocytes which renders them refractory to the tissue products which initiate inflammation. This is a possible explanation for the protective action of stress in delaying the onset of chemical carcinogenesis which was found in this department (Anderson 1964). When this observation is taken in conjunction with a previous finding of Anderson (1963) that obliteration of the draining lymph nodes about an area of chemical carcinogenesis by either thorotrast or local irradiation delays the onset of tumour formation, we are again led back to the cell and its surfaces.

It would seem probable, therefore, that the cell surface is implicated in carcinogenesis (Anderson and Green, 1963, 1965; Anderson, 1963, 1964) and that there is an infinitely complicated interplay between undefined locally acting forces and general circulatory regulators, both of which express themselves in, or on, the surface of the appropriate target cell.

This is, indeed, the logical conclusion to be drawn from Green's (1954) immunological theory of cancer in which he suggests that morphostasis is maintained by tissue specific antigen (TSA) and that when TSA is deleted or occluded by auto-antibody attack the cell becomes autonomous. It would seem that lost cell characteristics are not reconstituted at cell replication, but only at meiosis, and that the mutation in stem cells, as postulated by Burch, Burwell and Rowell,

(1965) is not an inevitable first step in carcinogenesis. If the lesion in carcinogenesis is indeed in, or on, the cell surfaces, the occasional regression of metastases after excision of the primary growth (Everson and Cole, 1956) also becomes more explicable.

SUMMARY

Consideration of normal and neoplastic cell surfaces makes it seem probable that the cell surface is implicated in carcinogenesis, and that lost cell characteristics are not reconstituted at cell replication.

### REFERENCES

Allison, F., Smith, M. R. and Wood, W. B.—(1955) *J. exp. Med.*, **102**, 669.

Anderson, M. R.—(1963) *Nature, Lond.*, **198**, 599—(1964) *Nature, Lond.*, **204**, 55.

Anderson, M. R. and Green, H. N.—(1963) *Nature, Lond.*, **198**, 861.—(1965) *Nature, Lond.*, **208**, 338.

Bangham, A. D., Rees, K. R. and Shotlander, V.—(1962) *Nature, Lond.*, **193**, 754.

Beiser, J. M., Erlanger, B. G., Agate, F. J. and Lieberman, S.—(1959) *Science, N.Y.*, **129**, 564.

Bullough, W. S.—(1966) *Cancer Res.*, **25**, 1684.

Burch, P. R. J., Burwell, R. G. and Rowell, N. R.—(1965) *Proc. R. Soc.*, B., **162**, 223, 240, 263.

Chambers, R. J.—(1922) *J. gen. Physiol.*, **5**, 189.

Coburn, A. F.—(1963) *Perspect. Biol. Med.*, **6**, 493.

Cohen, S. and Porter, R. R.—(1964) ‘ Advances in Immunology ’, edited by Dixon, F. J. and Humphrey, J. H. London and New York (Academic Press), p. 302.

Cook, G. M. W., Heard, D. H. and Seaman, G. V. F.—(1962) *Expl Cell Res.*, **28**, 27.

Cope, C. L.—(1965) ‘ Adrenal steroids and disease ’, edited by Cope, C. L. London (Pitman), p. 60.

Coutinho, E. M.—(1965) ‘ Hormonal Steroids ’, edited by Martini, L. and Pecile, A. London and New York (Academic Press), Vol. 2, p. 223.

Csapó, A.—(1956) *Recent Prog. Horm. Res.*, **12**, 405. (1960) in ‘ Structure and function of muscle ’, edited by Bourne, G. London and New York (Academic Press).

Desai, J. C. and Glover, J.—(1963) ‘ Biochemical problems of lipids ’, edited by Frazer, A. C. Amsterdam (Elsevier Press), Vol. 1, p. 44.

Dixon, M. and Webb, E. C.—(1958) ‘ Enzymes ’. London (Longmans Green).

Doljanski, F. and Eisenberg, S.—(1965) ‘ Cell electrophoresis ’, edited by Ambrose, E. J. London (A. & J. Churchill), p. 82.

Dourmashkin, R. R., Dougherty, R. M. and Harris, R. J. C.—(1962) *Nature, Lond.*, **194**, 1116.

Edelberg, R. J.—(1952) *J. cell. comp. Physiol.*, **40**, 529.

Engels, L. L.—(1959) *Vitams Horm.*, **17**, 205.

Erlanger, B. F., Borek, F., Beiser, S. M. and Lieberman, S. J.—(1957) *J. biol. Chem.*, **228**, 713.

Everson, T. C. and Cole, W. H.—(1956) *Ann. Surg.*, **144**, 366.

Forrester, J. A., Ambrose, E. J. and Macpherson, I.—(1962) *Nature, Lond.*, **196**, 1068.

Fuhrmann, G. F.—(1965) ‘ Cell electrophoresis ’, edited by Ambrose, E. J. London (Churchill), p. 92.

Gent, W. L. G., Trounce, J. R. and Walser, M.—(1964) *Archs Biochem. Biophys.*, **105**, 582.

Glick, J. L. and Githens, S.—(1965) *Nature, Lond.*, **208**, 88.

Green, H. N.—(1954) *Br. med. J.*, ii, 1378.

HEILBRUNN, L. V.—(1956) ' Dynamics of living protoplasm '. London and New York (Academic Press), p. 277.
HOTCHKISS, R. D.—(1944) *Adv. Enzymol.*, **4**, 187.
JACOBS, M. H.—(1962) *Circulation*, **26**, 1013.
JENSEN, E. V.—(1963) *Perspect. Biol. Med.*, **6**, 47.
KALCHER, H. M.—(1964) *Natn. Cancer Inst. Monogr.*, **14**, 21.
KIDSON, C. AND KIRBY, K. S.—(1964) *Nature, Lond.*, **203**, 599.
LIAO, S. AND WILLIAMS-ASHMAN, H. G.—(1962) *Proc. natn. Acad. Sci. U.S.A.*, **48**, 1956.
MCKINNELL, R. G.—(1962) *Amer. Zool.*, **2**, 430.
MICHAEL, R. P.—(1964) ' Hormonal steroids ', edited by Martini, L. and Pecile, A. London and New York (Academic Press), Vol. 2, p. 469.
MOLLER, E. —(1965) *Science, N.Y.*, **147**, 873.
MOVAT, H. Z. AND FERNANDO, N. V. P.—(1963) *Am. J. Path.*, **42**, 41.
NELSON, D. H., TANNEY, H., MESTMAN, J. H. AND GIESCHER, V.—(1964) ' Hormonal steroids ', edited by Martini, L. and Pecile, A. London and New York (Academic Press), Vol. 2, p. 563.
PULVERTAFT, R. J. V.—(1946) *J. clin. Path.*, **2**, 281.
RAPPORT, M. M. AND GRAF, L.—(1961) *Cancer Res.*, **21**, 1225.
RASMUSSIN, H., SCHWARTZ, I. L. AND SCHOESSLER, M. A.—(1962) *Proc. natn. Acad. Sci. U.S.A.*, **46**, 1278.
RIGGS, T. R.—(1964) ' Action of hormones on molecular processes ', edited by Litwack, G. and Kritchevsky, D. New York (Wiley), p. 1.
ROBERTSON, J. D.—(1960) *Prog. Biophys. biophys. Chem.*, **10**, 343.
ROSENBERG, M. D.—(1964) ' Cellular control mechanisms and cancer ', edited by Emmelot, P. and Mühlbock, O. Amsterdam (Elsevier Press), p. 146.
RUHENSTROTH-BAUER, G., FUHRMANN, G. F., GZANZER, E., KÜBLER, W. AND RUEFF, F.—(1962) *Naturwissenschaften*, **49**, 363.
SAWANT, P. L., DESAI, I. D. AND TAPPEL, A. L.—(1964) *Archs Biochem. Biophys.*, **105**, 247.
SCHULMAN, N.—(1965) ' Cell Electrophoresis ' edited by Ambrose, E. J. London (A. & J. Churchill), p. 82.
SCHWARZ, J.—(1959) *J. clin. Invest.*, **38**, 104.
SEKERIS, C. E. AND KARLSON, P.—(1964) *Archs Biochem. Biophys.*, **105**, 483.
SHAPRIR, E. AND KERPEL, S.—(1964) *Archs Biochem. Biophys.*, **105**, 237.
SLAUNWHITE, W. R. AND SANDBERG, A. A.—(1959) *J. clin. Invest.*, **38**, 1290.
TALALAY, P. AND WILLIAMS-ASHMAN, H. G.—(1960) *Recent Prog. Horm. Res.*, **16**, 1.
TALIAFERRO, I., COBEY, F. AND LEONE, L.—(1956) *Proc. Soc. exp. Biol. Med.*, **92**, 742.
TEPPERMAN, J. AND TEPPERMAN, H. M.—(1960) *Pharmac. Rev.*, **12**, 301.
VILLEE, C. A.—(1959) *Ann. N.Y. Acad. Sci.*, **75**, 524.
WALLACE, E. Z., SILVERBERG, H. I. AND CARTER, A. C.—(1957) *Proc. Soc. exp. Biol. Med.*, **95**, 805.
WEIR, D. M. AND PINCKARD, R. N.—(1965) *Lancet*, i, 1016.
WEISS, L.—(1965) *Expl Cell Res.*, **37**, 540.


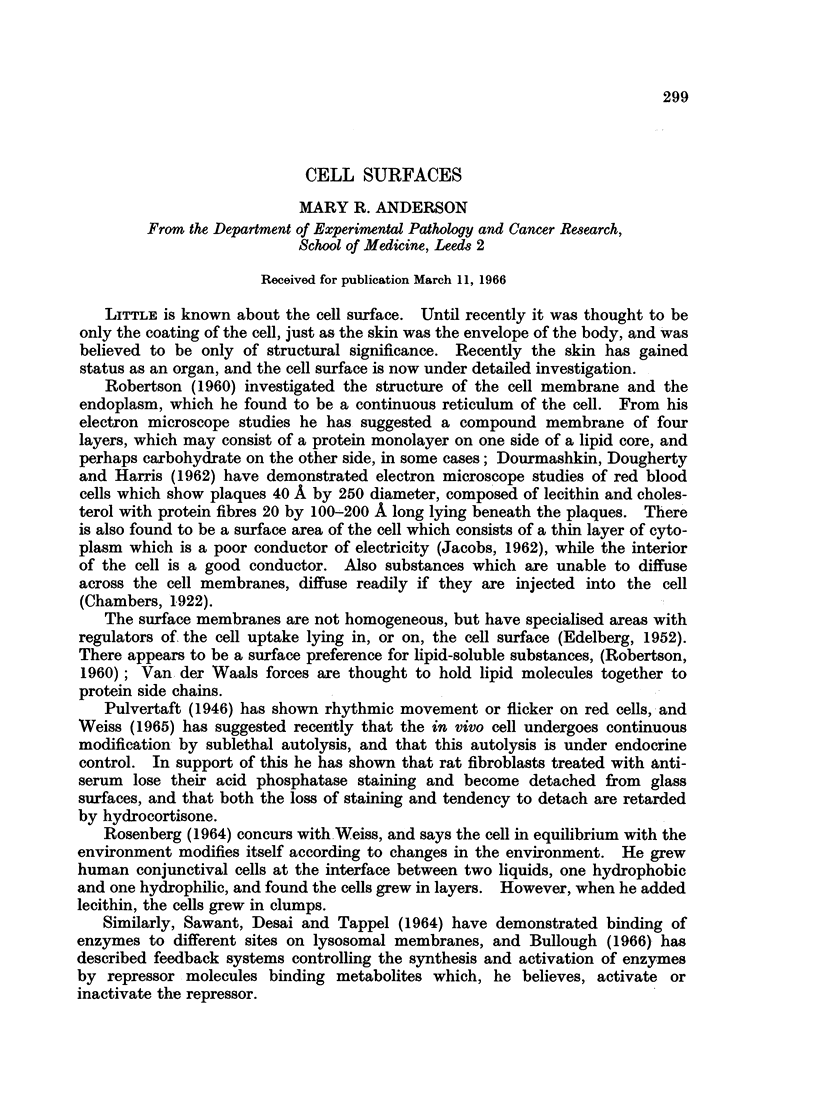

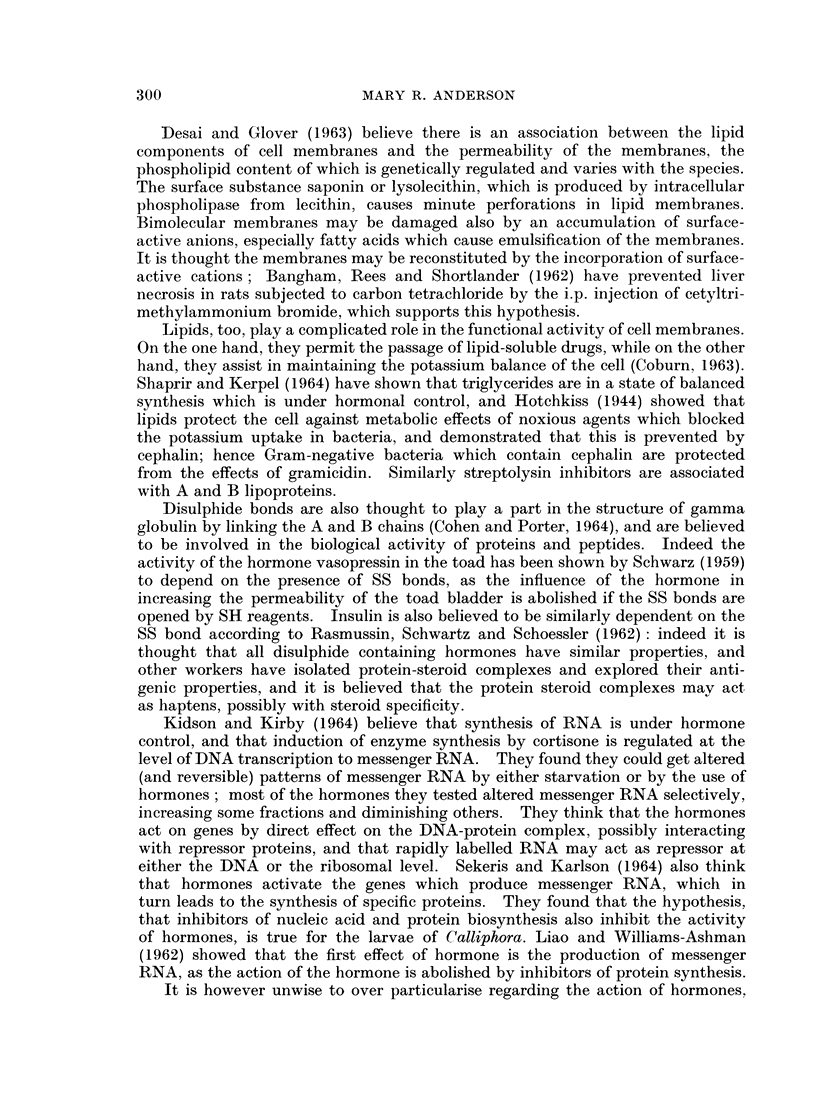

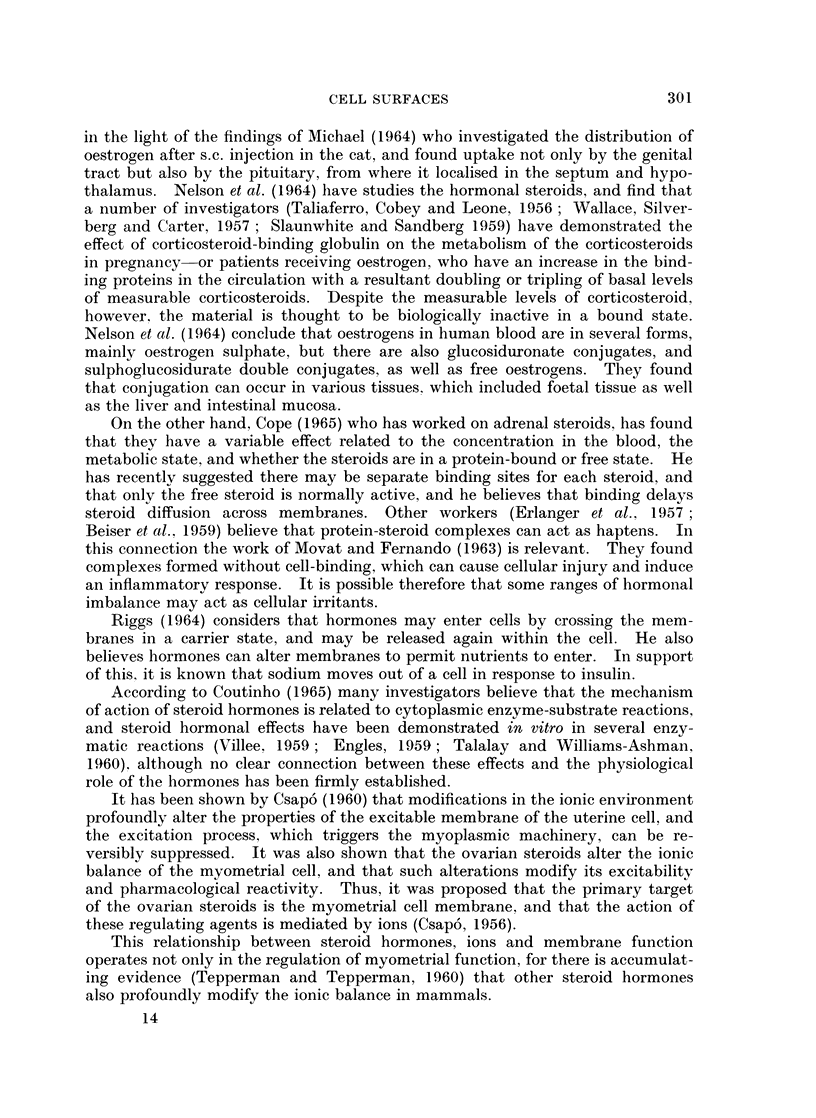

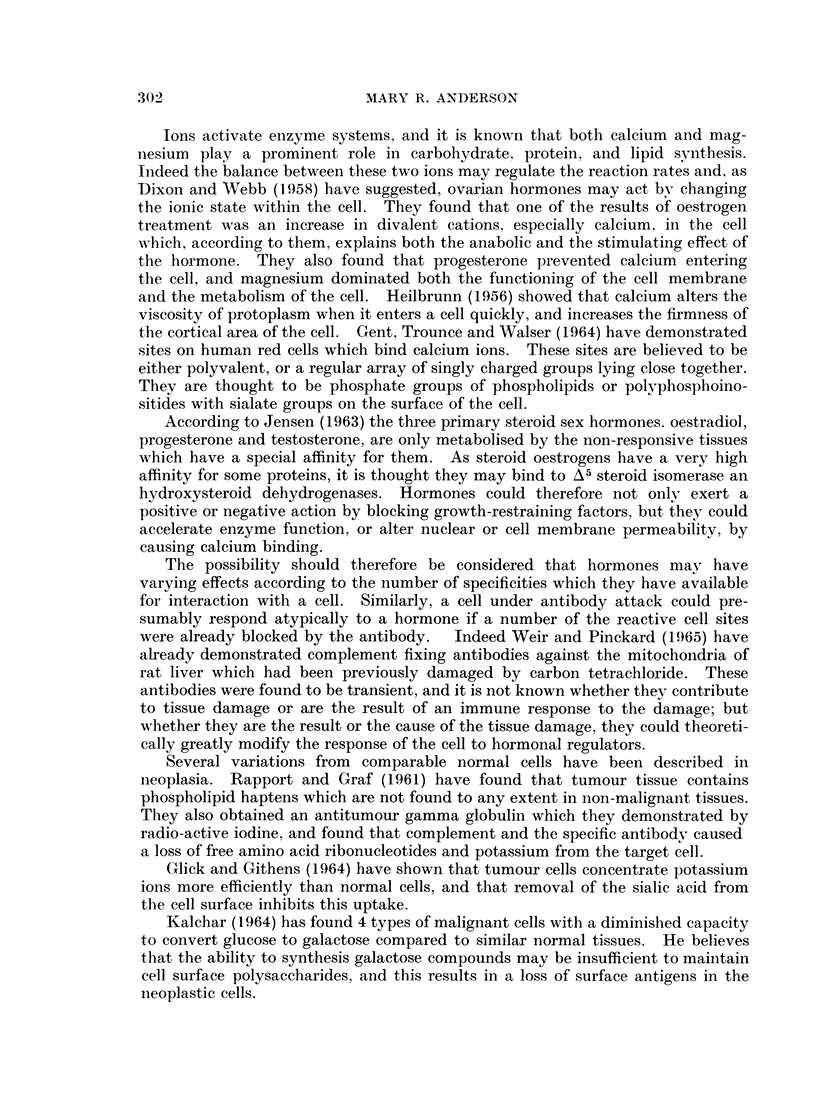

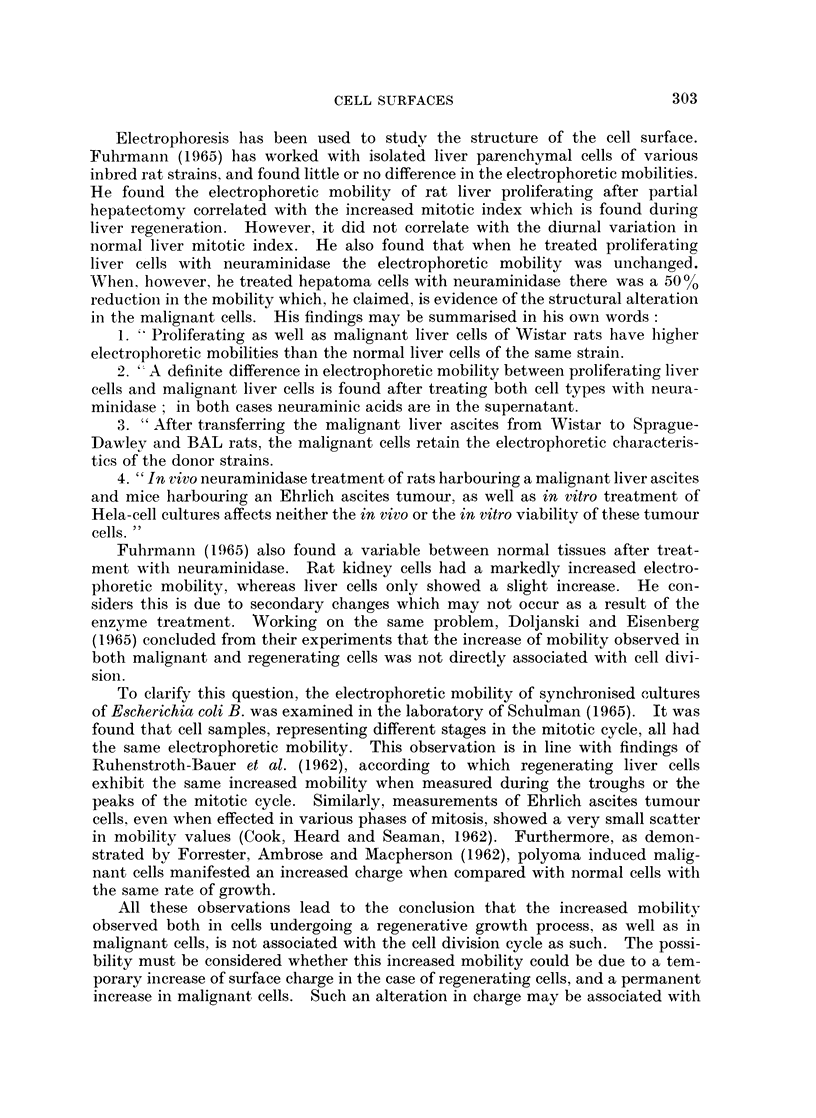

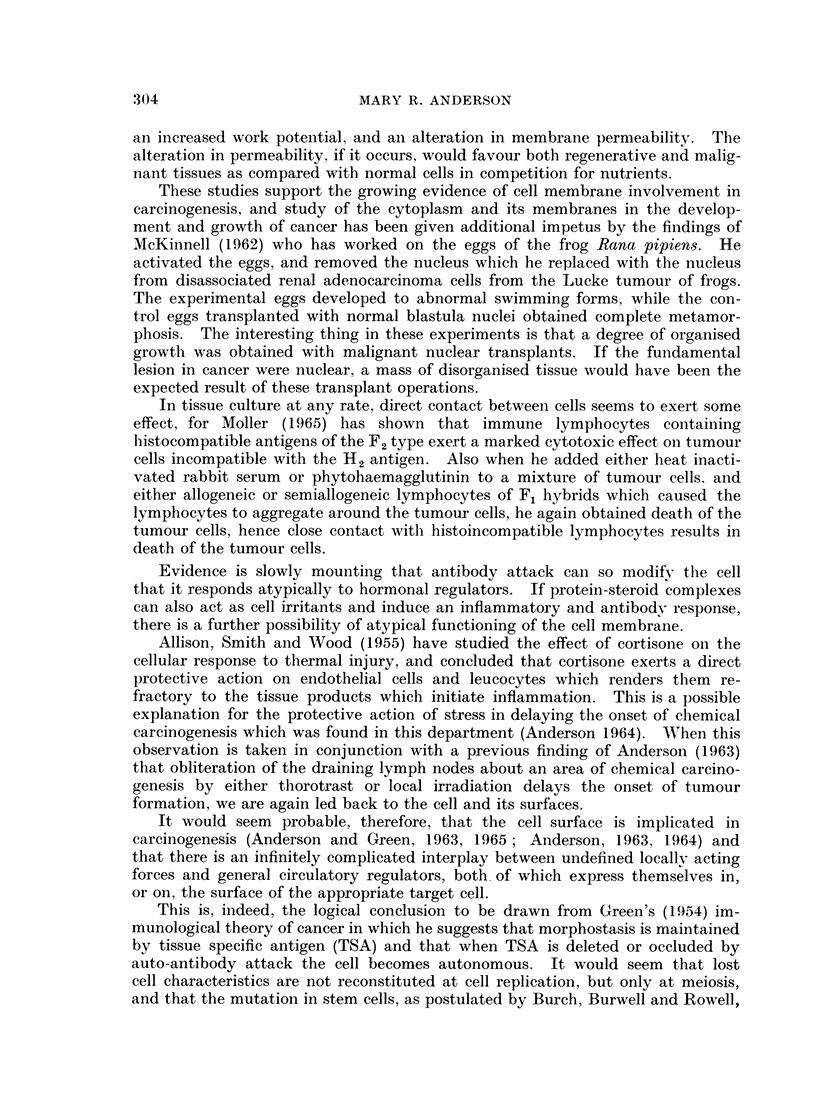

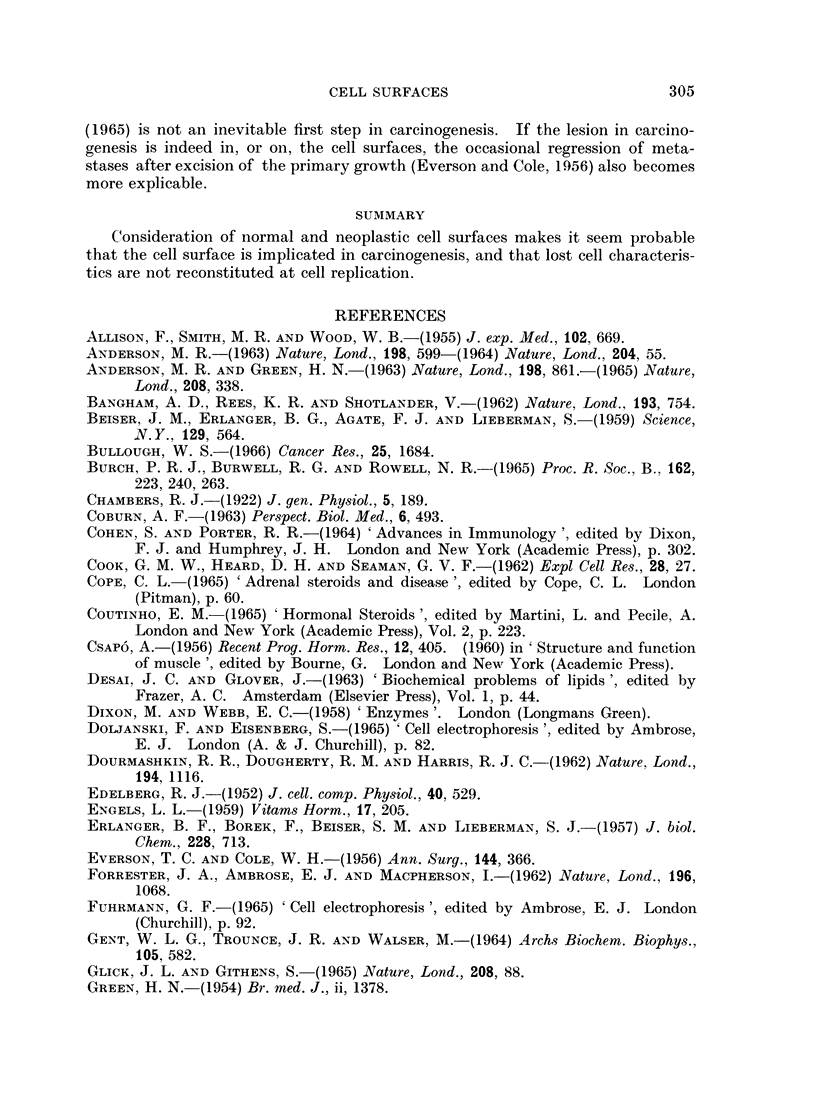

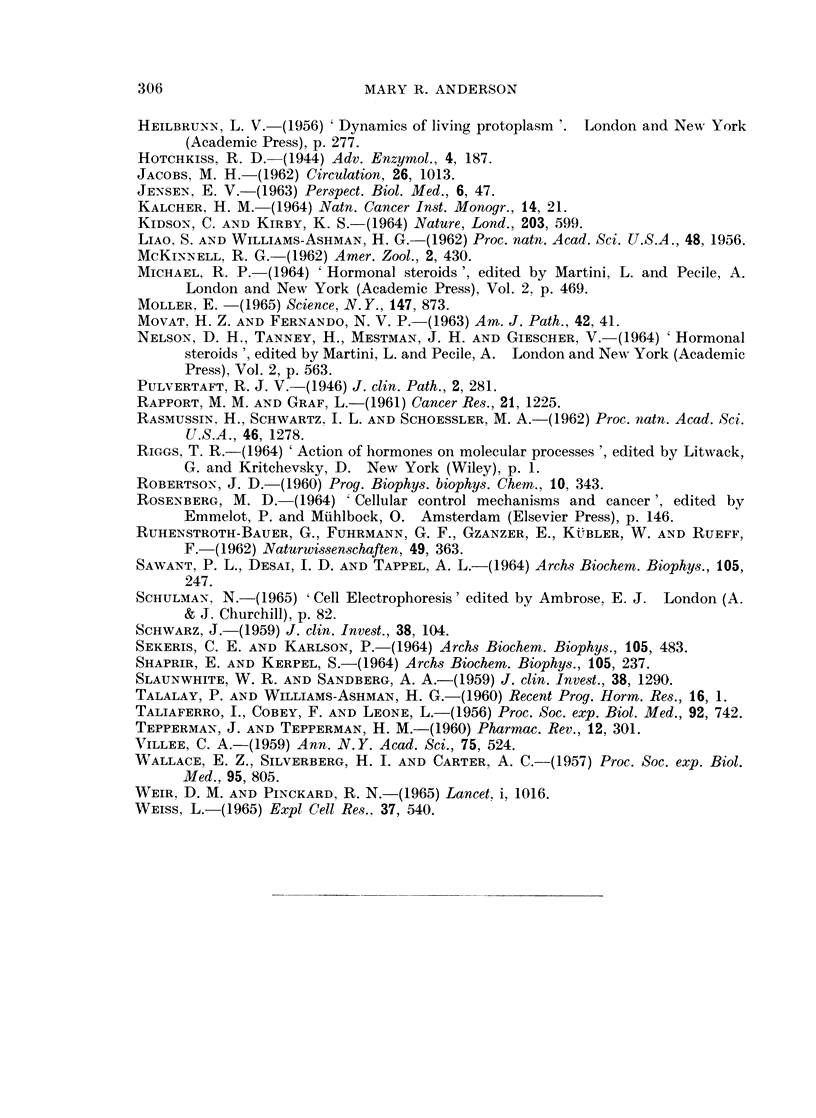


## References

[OCR_00379] ANDERSON M. R., GREEN H. N. (1963). Immunological implications of acquired tolerance to heterologous tumours.. Nature.

[OCR_00382] BEISER S. M., ERLANGER B. F., AGATE F. J., LIEBERMAN S. (1959). Antigenicity of steroid-protein conjugates.. Science.

[OCR_00388] BURCH P. R. (1965). NATURAL AND RADIATION CARCINOGENESIS IN MAN. II. NATURAL LEUKAEMOGENESIS: INITIATION.. Proc R Soc Lond B Biol Sci.

[OCR_00519] COBEY F., LEONE L., TALIAFERRO I. (1956). Effect of diethylstilbestrol on plasma 17-hydroxycorticosteroid levels in humans.. Proc Soc Exp Biol Med.

[OCR_00394] COBURN A. F. (1963). The pathogenesis of rheumatic fever--a concept.. Perspect Biol Med.

[OCR_00435] COLE W. H., EVERSON T. C. (1956). Spontaneous regression of cancer: preliminary report.. Ann Surg.

[OCR_00424] DOURMASHKIN R. R., DOUGHERTY R. M., HARRIS R. J. (1962). Electron microscopic observations on Rous sarcoma virus and cell membranes.. Nature.

[OCR_00427] EDELBERG R. (1952). The action of tannic acid on the erythrocyte membrane.. J Cell Physiol.

[OCR_00429] ERLANGER B. F., BOREK F., BEISER S. M., LIEBERMAN S. (1957). Steroid-protein conjugates. I. Preparation and characterization of conjugates of bovine serum albumin with testosterone and with cortisone.. J Biol Chem.

[OCR_00443] GENT W. L., TROUNCE J. R., WALSER M. (1964). THE BINDING OF CALCIUM ION BY THE HUMAN ERYTHROCYTE MEMBRANE.. Arch Biochem Biophys.

[OCR_00457] JACOBS M. H. (1962). Early osmotic history of the plasma membrane.. Circulation.

[OCR_00466] LIAO S., WILLIAMS-ASHMAN H. G. (1962). An effect of testosterone on amino acid incorporation by prostatic ribonucleoprotein particles.. Proc Natl Acad Sci U S A.

[OCR_00482] RAPPORT M. M., GRAF L. (1961). Cancer antigens: how specific should they be?. Cancer Res.

[OCR_00492] ROBERTSON J. D. (1960). The molecular structure and contact relationships of cell membranes.. Prog Biophys Mol Biol.

[OCR_00484] Rasmussen H., Schwartz I. L., Schoessler M. A., Hochster G. (1960). STUDIES ON THE MECHANISM OF ACTION OF VASOPRESSIN.. Proc Natl Acad Sci U S A.

[OCR_00515] SANDBERG A. A., SLAUNWHITE W. R. (1959). Transcortin: a corticosteroid-binding protein of plasma. II. Levels in various conditions and the effects of estrogens.. J Clin Invest.

[OCR_00520] TEPPERMAN J., TEPPERMAN H. M. (1960). Some effects of hormones on cells and cell constituents.. Pharmacol Rev.

[OCR_00521] VILLEE C. A. (1959). Estrogens and uterine enzymes.. Ann N Y Acad Sci.

[OCR_00523] WALLACE E. Z., SILVERBERG H. I., CARTER A. C. (1957). Effect of ethinyl estradiol on plasma 17-hydroxycorticosteroids, ACTH responsiveness and hydrocortisone clearance in man.. Proc Soc Exp Biol Med.

